# A Light-Weight Cropland Mapping Model Using Satellite Imagery

**DOI:** 10.3390/s23156729

**Published:** 2023-07-27

**Authors:** Maya Haj Hussain, Diaa Addeen Abuhani, Jowaria Khan, Mohamed ElMohandes, Imran Zualkernan, Tarig Ali

**Affiliations:** 1 Department of Computer Science and Engineering, American University of Sharjah, Sharjah P.O. Box 26666, United Arab Emirates; g00085636@aus.edu (M.H.H.); b00083108@aus.edu (M.E.); 2 Department of Civil Engineering, American University of Sharjah, Sharjah P.O. Box 26666, United Arab Emirates

**Keywords:** cropland extent, cropland intensity, machine learning, time series, Normalized Difference Vegetation Index

## Abstract

Many applications in agriculture as well as other related fields including natural resources, environment, health, and sustainability, depend on recent and reliable cropland maps. Cropland extent and intensity plays a critical input variable for the study of crop production and food security around the world. However, generating such variables manually is difficult, expensive, and time consuming. In this work, we discuss a cost effective, fast, and simple machine-learning-based approach to provide reliable cropland mapping model using satellite imagery. The study includes four test regions, namely Iran, Mozambique, Sri-Lanka, and Sudan, where Sentinel-2 satellite imagery were obtained with assigned NDVI scores. The solution presented in this paper discusses a complete pipeline including data collection, time series reconstruction, and cropland extent and crop intensity mapping using machine learning models. The approach proposed managed to achieve high accuracy results ranging between 0.92 and 0.98 across the four test regions at hand.

## 1. Introduction

Croplands are important not only for global food security but also for water security in a world where two-thirds of its population are estimated to experience severe water scarcity at least once a month [1,2]. According to the United Nations’ Food and Agriculture Organization (FAO) cropland is “land used for the cultivation of crops, both temporary (annuals) and permanent (perennials) and may include areas periodically left fallow or used as temporary pasture” [3]. Cropland mapping and global agriculture monitoring are therefore very essential for food security, agricultural resources management, agricultural economics, vegetation studies, and land use/land cover mapping purposes [4,5,6]. Croplands mapping and monitoring is a widely used application of remote sensing given its evident advantages of spatial coverage, cost effectiveness, monitoring ability for its revisiting frequency, and its ability to detect crops stress [7,8,9,10,11,12,13]. Crop intensity mapping, on the other hand, provides important information on the changes in and productivity of agricultural land [14]. Specifically, crop intensity mapping refers to the segmentation of agricultural land according to the number of crop planting cycles it exhibits, with cycle numbers ranging from 0 to 3, where 0 refers to no crops occurring, and 3 refers to the case in which the same land experienced 3 complete growth cycles of its crops in one year [15], [16]. Such a task plays an important role in improving food production, and agricultural planning, through surveying cropland changes [14,17].

Among vegetation indices, Normalized Difference Vegetation Index (NDVI) data are very popular remote sensing-based information for cropland coverage mapping and crops conditions assessment. NDVI is widely used to estimate the extent and vigor of vegetation on earth. NDVI is advantageous in defining vegetated areas, determining the biophysical conditions, including most vegetation phenological and physiological states, and in distinguishing between vegetation and bare soil. On the other hand, NDVIs major limitations are evident in very poorly or very highly vegetated areas. For instance, in areas with bare soil and very low vegetation cover, NDVI tends to be more sensitive to soil while in highly vegetated areas, NDVI tends to saturate. NDVI data have proven its robustness for cropland extent mapping [18], classification of agricultural lands [19], land suitability analysis for crop cultivation [20], crop type mapping [21], assessment of leaf area index (LAI), and vegetation fraction identification [22], to mention a few.

NDVI is typically estimated using the percentage surface reflectance values of the red and near-infrared (NIR) bands acquired through remote sensing. NDVI is a unitless index that ranges from −1 to +1, with values close to −1 indicating areas with no vegetation or water, values close to 0 indicating areas with sparse vegetation or bare soil, and values close to +1 indicating areas with dense and healthy vegetation.

The availability of remote sensing data from different satellite programs and the ease of access to such data have led to the availability of time series NDVI datasets on global, regional, and local scales. Some of the satellite programs that make data freely available to the end users include MODIS, Landsat, and Sentinel, along with limited commercial satellite data with higher spatial and temporal resolutions from programs such as WorldView-3,-4, and Planet [23].

The NDVI time series data, however, typically contain some errors due to atmosphere conditions, cloud cover, and sensor capacity. It is therefore very important to analyze, smooth, and reconstruct NDVI data before its use in cropland mapping [24,25,26]. Matsushita et al. [27] and Kumari et al. [28] have discussed the effect of topographic illumination, shading effects, and solar angle issues on NDVI. Kumari et al. [28] has provided a better understanding of the variations of vegetation on hillslopes facing opposite directions. The study has shown that variations of solar radiation on polar-facing slopes would lead to higher values of NDVI compared to equatorial-facing slopes. Further, a discussion by Kumari et al. [28] has highlighted the advantage of the band rationing of NDVI in eliminating most of the effect topographic illumination, shading effects, and solar angle issues [28,29]. The common NDVI data reconstruction techniques for local and regional studies are spatial and based on proximity analysis. These methods consider the correlation between pixels and their neighborhood to restore the missing values. Some of the common spatial methods are based on linear, bilinear, and kriging interpolation. Some of the popular methods for reconstructing NDVI time series data are temporal- or spatiotemporal-based, which can generally be categorized into temporal interpolation, temporal filtering, temporal function-fitting, temporal deep learning, frequency-based methods, and hybrid techniques [30,31,32].

Given their high computational speed and promising classification results, many machine learning and AI techniques have been used with remote sensing data for the mapping and monitoring of cropland. Some of the popular methods include random forest, neural networks, and support vector machines [33,34,35,36]. These algorithms typically learn about the target class characteristics from the training dataset and identify the various classes in the input dataset. The recent literature on this topic includes a study by Ketchum et al. [37], in which a method for the large-scale mapping of irrigated agricultural lands in Western U.S. using random forest machine learning was developed. Zhang et al. [38] have mapped croplands in China with a machine learning classifier on Google Earth Engine.

Conducted as part of the International Telecommunication Union (ITU) competition, the main objective of this study was to develop a robust, cost-effective, machine learning, and AI-based method for cropland and crop intensity mapping in four study areas: Iran, Mozambique, Sri Lanka, and Sudan. The competition tasks included training dataset creation, NDVI data time series reconstruction, and cropland extent mapping as well as crop intensity mapping. The contributions of this paper are summarized in the following points:

1. Provide a detailed methodology of data points collection of Sentinel-2 satellite assets though google earth engine for cropland extent and intensity applications for machine learning.
2. Perform a NDVI time-series reconstruction on the data-points collected to patch gaps and errors within the series using a Savitzky–Golay filter and linear interpolation technique.
3. Develop an adaptive threshold approach for crop intensity cycles detection based on the obtained NDVI time-series.
4. Apply different machine learning techniques on the dataset at hand to generate cropland extent and intensity maps.
5. Compare context aware and regular machine learning techniques in terms of efficiency and speed.

## 2. Materials and Methods

### 2.1. Sentinel-2 Data

Sentinel-2 data were used as input for our cropland extent and intensity models. The European Space Agency oversaw the development of the Sentinel-2, which is a constellation of two Earth observation satellites, as a part of the Copernicus Earth observation program of the European Commission. Sentinel-2 satellites’ wide-swath, multi-spectral imaging capabilities offer an unprecedented perspective of the Earth, encompassing all its landmasses, sizable islands, and waterways. Applications in forestry, agriculture, and other land management fields benefit greatly from Sentinel-2 data. For instance, it can be used to map forest cover and soils, investigate leaf area together with chlorophyll and water content, and monitor inland waterways and coastal regions [39]. Two identical satellites make up the Sentinel-2 mission: Sentinel-2A, which was launched on 23 June 2015, and Sentinel-2B, which was launched in 2017. The constellation can revisit each location on the surface of the Planet once every five days with both satellites launched. Each satellite is equipped with a Multi-Spectral Instrument (MSI), which creates photographs of the Earth with a resolution of ten meters per pixel, a field of view of 290 km, and thirteen bands spanning the visible and infrared spectrum [39].

### 2.2. Study Area and Dataset

**Figure 1:**
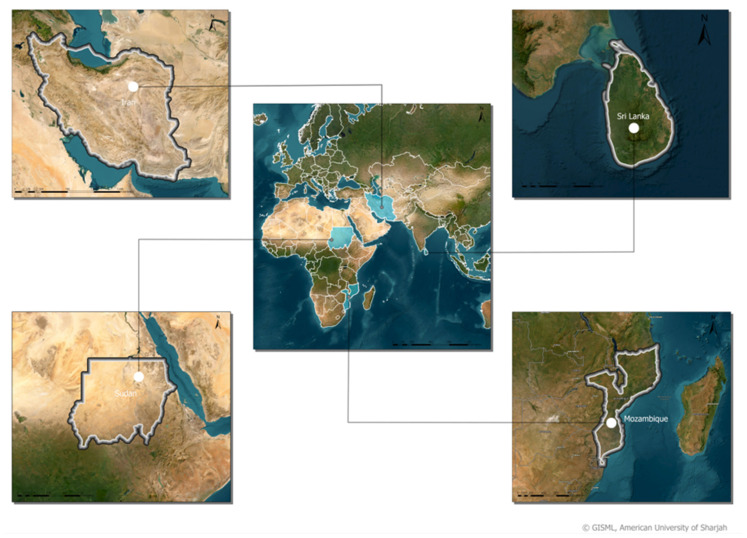
Overview of the four study regions addressed in this study which includes Iran, Sri-Lanka, Sudan, and Mozambique (clockwise).

The study region of this paper covers four different countries: Iran, Sudan, Mozambique, and Sri Lanka, as illustrated in Figure 1. The regions covered are fairly diverse in land cover types, including grassland, forests, water, bare soil, and asphalt; but, most importantly, they include ample agricultural land. The four regions differ in climate, with the region in Iran having a semi-arid climate, the region in Sudan having a hot desert climate, and lastly, the region in both Mozambique and Sri Lanka having a tropical climate. Consequently, the cropland captured in the study area’s collected data is diverse in the type of crops included.

The datasets used in this paper consist of Normalized Difference Vegetation Index (NDVI) time series data of 10-m spatial resolution collected from a 15-day Sentinel-2 composite, which covers a region of 0.5 degrees by 0.5 degrees for each of the four studied countries. NDVI is a remote sensing index used to assess vegetation health and quantity [40]. NDVI also helps differentiate vegetated land from other land-cover types in imagery as it tends to have a positive value for pixels related to vegetated land and zero or a negative value for pixels pertaining to other types of land-cover, such as water [41]. Hence, NDVI was used in this work in order to perform both cropland and crop intensity mapping. NDVI relies on the red band and the Near-Infrared band and is calculated as follows, where NIR and red are the surface reflectance valeus of the Near-Infrared band and the Red band respectively:

(1) $$\mathrm{NDVI}=\frac{\mathrm{NIR}-\mathrm{Red}}{\mathrm{NIR}+\mathrm{Red}}$$

The geo-spatial assets were provided by the international AI for good and ITU Cropland mapping competition organizers. The exact type of crops mapped is relative to the study region. The tiles provided were later used to generate data points using Google earth engine by applying two different data collection approaches based on the problem requirement. For the problem of cropland extent, we collected 75 geometric points of cropland and 75 points of non-cropland using eye inspection for each of the four study regions. The geometric points were then appointed a binary label according to their class (cropland, non-cropland). As for the problem of crop intensity mapping, we used geometric polygons of water surfaces, cropland, and non-cropland areas to form our datasets. The polygons allowed us to collect many data points, ensuring that we captured data pertaining to all crop intensity classes, of which we only used 200 points. These data points were then appointed a label of zero crop cycles, one crop cycle, two crop cycles, or three crop cycles through the crop cycle counting algorithm described later in this paper with the crop cycle number referring to the number of complete crop growth cycles a land exhibited in one year. After appointing the labels, we dealt with any imbalances found in the datasets caused by under-representation of certain classes by oversampling these underrepresented classes. It is worth mentioning that the size of the datasets was restricted to a maximum of 500 training data points per dataset as per competition rules, with smaller datasets being preferred, hence the small size of the collected datasets for this paper. Each of these data-points represented a pixel-wise NDVI value recorded every two weeks which resulted in a time-series of 24 points in total.

### 2.3. Data Pre-Processing

This section details the dataset pre-processing and preparation procedure followed in this paper.

#### 2.3.1. Cropland Extent Samples

**Figure 2:**
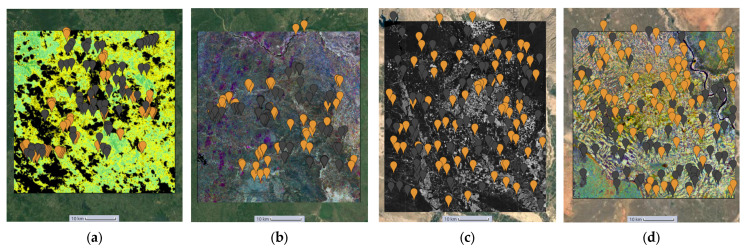
(**a**) Sri-Lanka cropland extent samples. (**b**) Mozambique cropland extent samples. (**c**) Iran cropland extent samples. (**d**) Sudan cropland extent samples.

Figure 2 shows the four study areas data collection for the cropland extent problem with 150 samples per country. Those 150 samples were divided into 75 cropland and 75 noncroplands. These samples are visualized in the figures below, with orange pins representing cropland areas and grey pins representing non-cropland areas.

#### 2.3.2. Cropland Intensity Samples

**Figure 3:**
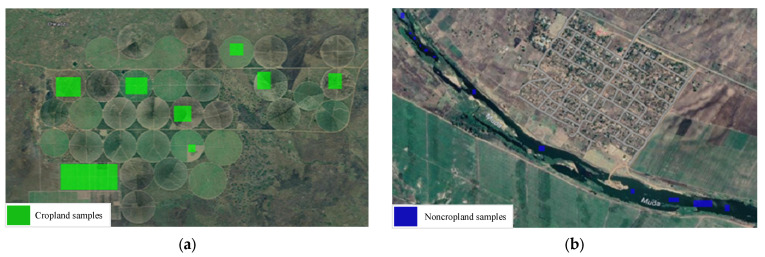

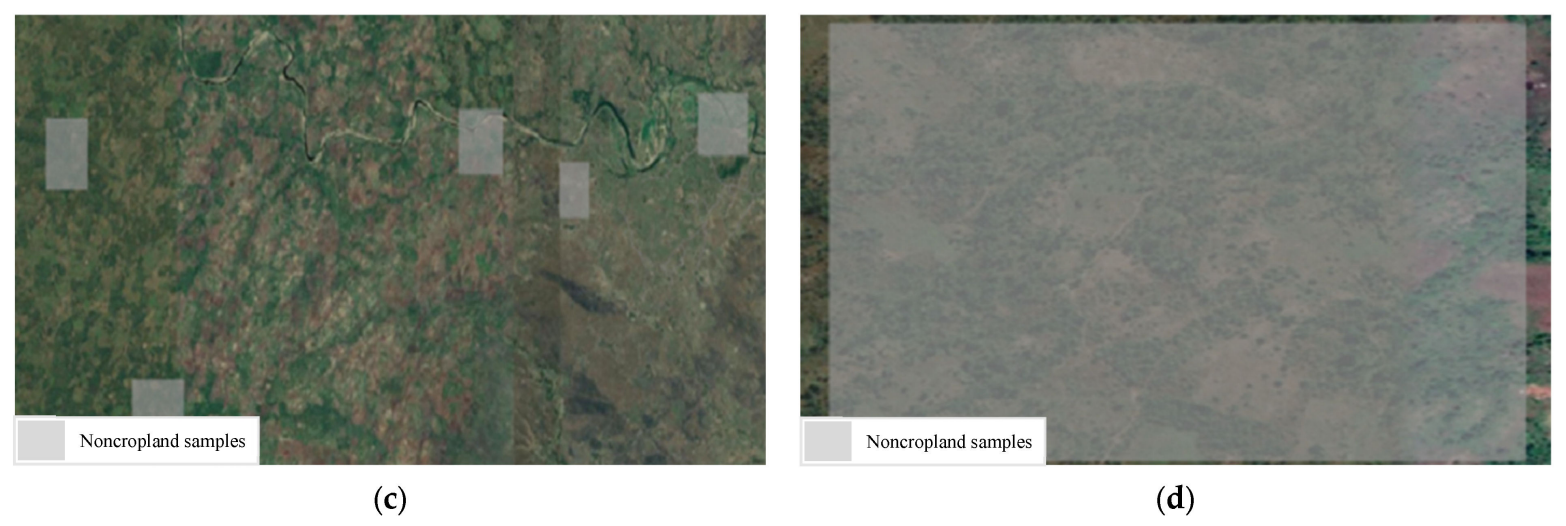
(**a**) Mozambique cropland samples. (**b**) Mozambique water samples. (**c**) Mozambique noncropland samples. (**d**) Mozambique noncropland samples zoomed in.

Figure 3 illustrates the data collection process for the cropland intensity problem in Mozambique. This process was replicated for the three other study areas. Samples were collected using polygons to ensure comprehensive coverage of all four crop intensity classes and to guarantee sample variability within each class. 

#### 2.3.3. Cropland Extent and Intensity Samples Collection Procedure

**Figure 4:**
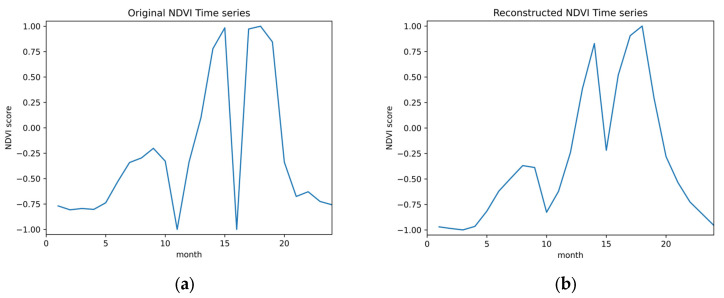
(**a**) NDVI time-series before reconstruction (**b**) NDVI time-series after reconstruction.

**Figure 5:**
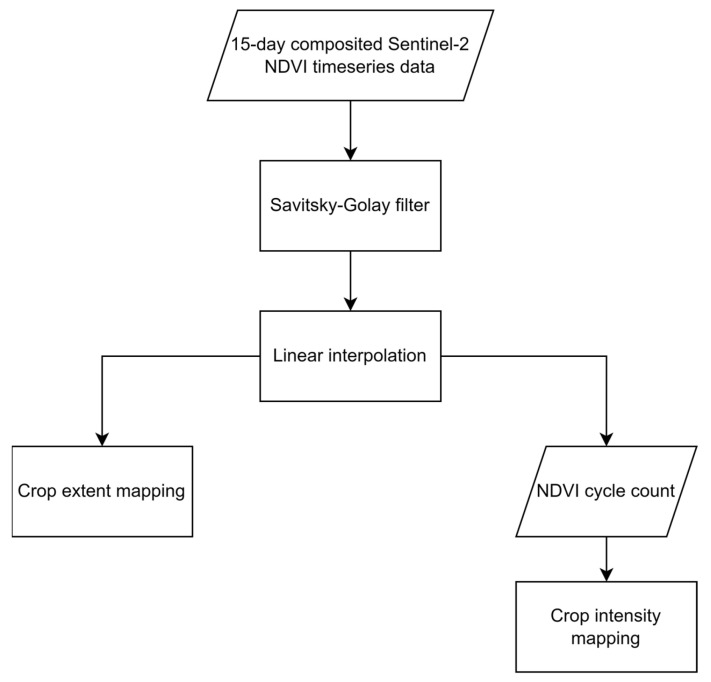
The flowchart of the dataset pre-processing and preparation procedure.

The dataset used for the two problems of cropland extent and intensity mapping was collected from a gap-filled NDVI time series provided by the ITU competition organizers. Since training classifiers on an incomplete time-series negatively impacts their accuracy, it was necessary to first reconstruct the dataset’s time series data, filling in the gaps where data were missing, before proceeding to use the dataset to train any classifier. Hence, to fill in the gaps, iterative interpolation for data reconstruction (IDR) and was used and missing data points were simply replaced with the average value of their adjacent points where sudden and drastic changes in NDVI values were noted. Further, linear interpolation was also applied when an NDVI value difference greater than 0.4, around half of the greatest NDVI value found in the collected dataset, was noted between two adjacent points where the second point was replaced to achieve a more natural rate of change in the NDVI scores of the time series data. This method was selected as the gap-filling procedure amongst other more complex options such as Fourier-based approach (Fourier), the double logistic model (DL), the Whittaker smoother (Whit), and the locally adjusted cubic spline capping approach (LACC) [42] given that the missing NDVI values were not expected to drastically deviate from their neighboring values, that is, following a typical vegetation growth cycle, and that such a replacement was not going to affect the mapping for which the classifiers are training. The time-series was further cleaned by applying a quadratic-polynomial-based Savitzky–Golay filter to the collected time series data, resulting in smoother NDVI time series curves. Given that the dataset’s time series curves contained 24 points only, the filter was configured to have a sliding window of 3 points to not cause significant loss in the time series’ curve characteristics. Figure 4 shows a sample time series from the collected dataset before (a) and after (b) applying the detailed reconstruction method. The flowchart in Figure 5 depicts said procedure.

### 2.4. Adaptive Threshold Approach for Crop Intensity Cycles Detection

While labelling for the cropland extent dataset was performed through visual interpretation, crop intensity labels were not determined as easily. Crop intensity was instead determined through an algorithm developed for the purpose of crop cycle counting. The developed algorithm traversed each time series in the dataset and found the total number of peaks in the time series curves. Based on the number of peaks found, a time series was assigned a label: A class 0 label when no crop cycles were detected, a class 1 label when only one crop cycle was detected, a class 2 label when two crop cycles were detected, and lastly, a class 3 label when three crop cycles were detected.

The developed algorithm paid particular attention to eliminating spurious peaks from the crop cycle count. Spurious peaks mainly occur in time series data due to the growth of weeds in fields, which cause spikes in NDVI scores. These spikes are presented in NDVI time series curves as small peaks with values, much lower than that of cropland peaks which have NDVI scores typically above 0.5 [43]. When these peaks are not dealt with, the crop cycle count in a given time series is overestimated and hence the resulting crop intensity estimate made is inaccurate. The algorithm first traverses a given time series and finds the maximum NDVI score. If the highest NDVI score in the time series is found to be less than 0.3, the time series is immediately given the class 0 label, meaning it does not contain any crop cycle. This is because NDVI timeseries of such low NDVI scores cannot correspond with actively used cropland, which typically reflect higher NDVI scores, instead they are more likely to pertain to barren land, rocks, and water or sparse vegetation. Otherwise, two thresholds are created based on this value: a minimum value for thresholding peaks and a maximum value for valleys. These thresholds are used to find the number of crop cycles in each time series while making sure that spurious peaks caused by weeds are not included in the count. In the previous literature relating to crop intensity mapping, spurious peaks were removed from the crop cycle count by simply using a static threshold for the peak values, where peaks that fell below a specific NDVI or EVI score were considered spurious [15,43,44]. However, we have found that such an algorithm is insensitive to the fact that different types of crops differ in the NDVI scores they can attain during a crop cycle, assuming one threshold for all types of crops. This insensitivity can result in underestimating crop intensity. To deal with this problem, we introduced the concept of adaptive thresholds, where peaks and valleys in a given time series are accepted based on thresholding values tailored to that time series. In the case of our algorithm, we set the thresholding value for peaks in any time series to be 0.70 of the NDVI score of the absolute maxima in that time series, whereas the valley threshold was set to be 0.20 of that value. After finding the thresholds, the time series is traversed once more in order to find the number of crop cycles. A crop cycle is then considered to have occurred when a peak value that is encompassed between two valley values is found. In the special case where the peak value is found at an endpoint, a crop cycle is counted if and only if the peak is either lead or followed by a valley.

### 2.5. Classification Methods

There are primarily five machine learning algorithms deployed for the purpose of the binary classification of our sample points for the cropland extent problem as well as the categorical classification of our reconstructed NDVI time series for the crop intensity problem. The models used for this purpose are as follows:

#### 2.5.1. Random Forest

Random forest is a classification algorithm that is built of multiple decision tress. Firstly, n records are selected at random from a data set with k records. Next, each sample’s decision tree is built separately, and consequently, each decision tree produces an output. Finally, the model’s output is based on the Majority Voting ensemble that combines the results from all the individual decision trees [45].

#### 2.5.2. XGBoost Classifier

XGBoost is short for Extreme Gradient Boosting. It is a distributed gradient-boosted decision tree. The trees are boosted by parallel trees and are usually the leading machine learning algorithm for classification problems. Similar to random forest, XGBoost also employs decision trees as base learners. However, the trees used by XGBoost are CARTs (Classification and Regression trees) that contain real-value scores in each leaf node instead of a single decision [46].

#### 2.5.3. LSTM

LSTM [47] is short for Long Short-Term Memory and is a special type of RNN (Recurrent Neural Network) capable of handling the vanishing gradient issue faced by RNN. It can process the entire sequence of data and remember long term dependencies. LSTM has feedback and has an input flow which can either be backwards or forwards. Equations (2) and (3) describe the encoder and decoder of an LSTM; where $h_{t}$ is the encoder’s hidden state at time step t with input token embedding $x_{t}$. On the decoder side, $s_{t}$ denotes the hidden state at time step t with input embedding token $y_{t}$. In this study, we have constructed an LSTM model consisting of one LSTM layer of 50 units, followed by a dense layer.

(2) $$h_{t}={\mathrm{RNN}}_{enc}\left(x_{t},h_{t-1}\right)$$

(3) $$s_{t}={\mathrm{RNN}}_{dec}\left(y_{t},s_{t-1}\right)$$

#### 2.5.4. Bidirectional LSTM

Bidirectional LSTM works similar to LSTM, but the main difference is that bidirectional can make input flows in both directions, backwards and forward, since it consists of two LSTMs: one which takes the input in the forward direction and the other taking it in the backward direction. With the help of BiLSTMs, the network has access to more information, which improves the context available to the algorithm [48]. In our study, we have constructed a Bi-LSTM model comprising two consecutive BiLSTM layers of 50 units each, followed by a dense layer.

#### 2.5.5. KNN DTW

KNN is the K-nearest neighbors’ algorithm. It is well known for classification and works by finding the distances between a value and other examples in the data and then chooses the most frequent label for classification. DTW stands for Dynamic Time Warping and is used in time series analysis. It measures the similarity between two temporal sequences. This approach is popular for time series classification given the algorithm’s speed and scalability. Since we are dealing with the crop intensity problem involving NDVI time series classification, KNN with DTW seemed to be the more appropriate choice of algorithm for this specific problem [49].

#### 2.5.6. Computational Complexity

As we are looking into developing a light-weight solution to cropland mapping, accuracy is not the only metric that should be taken into consideration, the computational complexity of the algorithms implemented should also be considered. In general, traditional machine learning algorithms are more efficient than ensemble methods and recurrent neural networks. The complexity of Random forest is the lowest among tested algorithms with a training time complexity of $O\left(T\times n\times log\left(n\right)\times m\right)$ and a space complexity as small as $O\left(d\times k\right)$ where T is the number of trees, n is the number of training samples, m is the number of features, d is the depth of tree, and k is the number of neighbors [50]. On the other hand, KNN-DTW follows a slightly more expensive algorithm with a training time complexity of $O\left(k\times n\times m\right)$ and a space complexity of $O\left(n\times m\right)$ [51] while XGBoost follows a lower efficiency level of $O\left(Kd\left||x\right|{|}_{0}logn\right)$ [52]. Finally, the time complexity of LSTM and Bidirectional LSTM relies on the number of edges in the network W with a time complexity of $O\left(W\right)$ and $O\left(2W\right)$, respectively [53]. In general, literature suggests that there is a tradeoff between accuracy and speed when it comes to generating cropland maps. Traditional machine learning algorithms are not as computationally expensive as RNNs and are thereby faster but have slightly less accurate results.

## 3. Results and Discussion

Our results in Table 1 show that traditional machine learning algorithms, such as Random Forest and XGBoost, performed well across all four test regions for cropland extent. XGBoost model achieved the highest average accuracy (88.5%) overall outperforming more computationally expensive recurrent neural network models such as LSTM and Bidirectional LSTM. In general, the test region of Iran resulted in the best performance where Bi-LSTM achieved 98% accuracy. On the other hand, Mozambique’s test region was the most challenging as the highest accuracy achieved was 82% through both XGBoost and LSTM models. 

Table 2 shows that the results obtained for cropland intensity mapping problem were consistent with those of cropland extent mapping, as XGBoost scored the highest average accuracy (87.3%) across all test regions and outperformed random forest model and KNN DTW model. Context aware models such as LSTM, Bidirectional LSTM, and KNN DTW seemed to perform better than context unaware algorithms.

### Maps Generated as Google Earth Engine Assets

**Figure 6:**
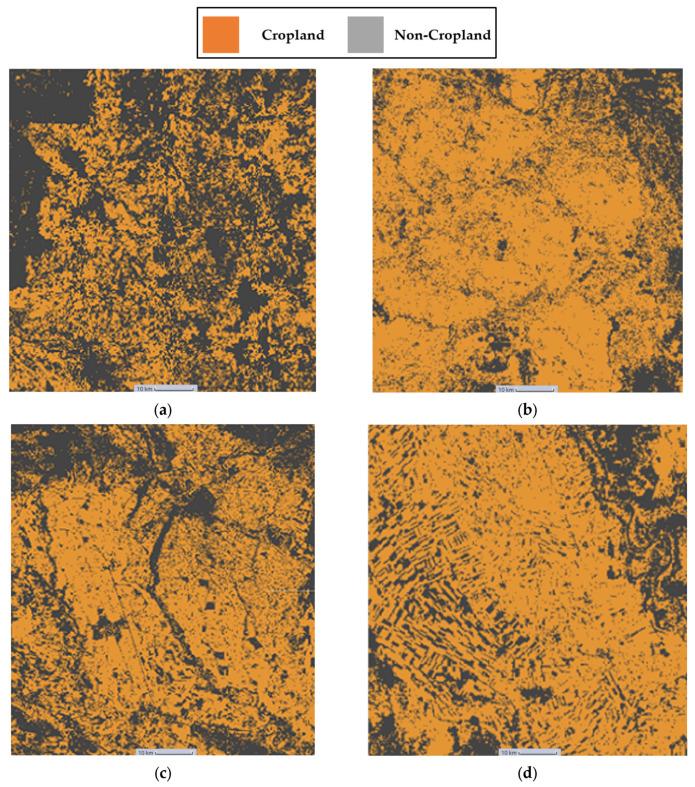
(**a**) Sri-Lanka cropland extent result map. (**b**) Mozambique cropland extent result map. (**c**) Iran cropland extent result map (**d**) Sudan cropland extent result map.

**Figure 7:**
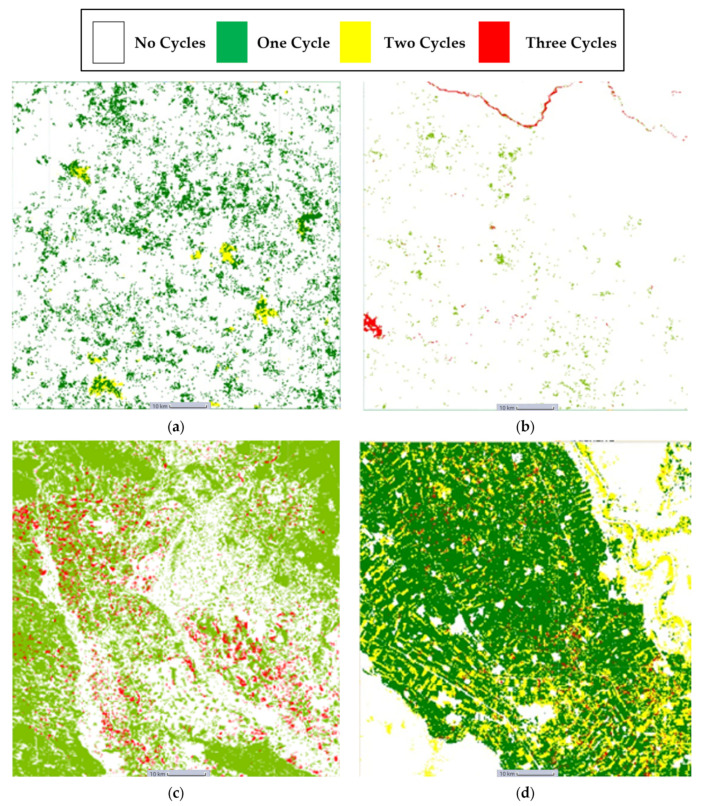
(**a**) Sri-Lanka cropland intensity result map. (**b**) Mozambique cropland intensity result map. (**c**) Iran cropland intensity result map (**d**) Sudan cropland intensity result map.

These results can be explained by the geographical nature of the four test regions. We believe that the presence of many forests in the region of Sri-Lanka (Figure 6a and Figure 7a) reduced the model’s performance as the corresponding time series of forests samples were like those of cropland areas. The region of Mozambique (Figure 6b and Figure 7b) contained samples that were difficult to distinguish even through eye-inspection, while the region of Iran (Figure 6c and Figure 7c) contained easily distinguished cropland or non-cropland areas with a high variance present in the corresponding time series. Additionally, the presence of a body of water such as the Nile River in the test region of Sudan (Figure 6d and Figure 7d) could decrease the performance because it is fringed by gallery forests and herbaceous vegetation which can be misleading.

To test the inference time of the algorithms at hand, we measured the time needed to generalize the results on a 0.5° by 0.5° grid, which corresponds to about 55 km squared, in the four test regions.

As Table 3 shows, traditional machine learning methods seemed to be more time-efficient when applied on the full map with an average inference time of 4 min for Random Forest and less than a minute using XGBoost with a Python 3 Google Compute Engine backend. On the other hand, context aware models took longer time to perform the same task with approximately 1 h 30 min for the LSTM model using the same computational resources. These results are consistent with the theoretical computational complexity analysis of the algorithms discussed earlier. 

**Figure 8:**
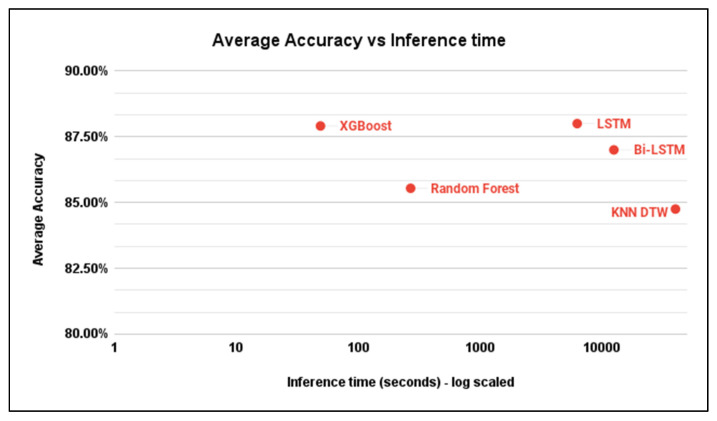
Inference time of different models on a 0.5 degree by 0.5 degrees grid size 4.

We believe that traditional machine learning algorithms are the optimum solution to operate on large grid maps given their relatively high accuracy, low computational cost, and fast inference time. Figure 8 illustrates the average inference time of each model with respect to the average accuracy. 

## 4. Conclusions

In this study, we present a comprehensive pipeline for cropland extent and intensity mapping using lightweight machine-learning models. Time series samples were collected from Sentinel-2 data using Google Earth Engine and utilized to train various machine learning models for cropland and crop intensity classification after a series of preprocessing steps. The performance of traditional machine learning algorithms, such as Random Forest and XGBoost, was compared with context-aware methods like KNN-DTW and with Recurrent Neural Networks (LSTMs and Bi-LSTMs). The evaluation results of this study demonstrate that all algorithms achieved high accuracy, ranging between 84% and 98%, across four test regions: Iran, Sri Lanka, Sudan, and Mozambique. After evaluation, the obtained results were generalized over a complete map with a grid size of 0.5° by 0.5°, captured from 10-m spatial resolution data, after which the inference time of each algorithm was measured, and all models were compared. Based on our findings, we conclude that traditional machine learning algorithms offer a more efficient and lightweight solution for cropland mapping due to their relatively high accuracy and fast inference time.

## Figures and Tables

**Table 1:** Cropland Extent Results (Average Accuracy).

Model	Mozambique	Sudan	Iran	Sri-Lanka
Random Forest	0.74	0.84	0.92	0.86
XGBoost	0.82	0.92	0.92	0.88
LSTM	0.82	0.84	0.96	0.90
Bidirectional LSTM	0.80	0.90	0.98	0.80

**Table 2:** Cropland Intensity Results.

Model	Mozambique	Sudan	Iran	Sri-Lanka
Random Forest	0.87	0.86	0.803	0.95
XGBoost	0.92	0.83	0.803	0.94
LSTM	0.75	0.92	0.80	0.92

**Table 3:** Average inference time using different machine learning algorithms.

Model	Average Inference Time (Seconds)
Random Forest	49
XGBoost	269
LSTM	6259
Bi-LSTM	12,518
KNN DTW	40,065

## Data Availability

No new data were created or analyzed in this study. Data sharing is not applicable to this article.

## References

[1] Summary Progress Update 2021: SDG 6—Water and Sanitation for All. https://www.unwater.org/publications/summary-progress-update-2021-sdg-6-water-and-sanitation-all.

[2] Thenkabail P.S. (2010). Global Croplands and their Importance for Water and Food Security in the Twenty-first Century: Towards an Ever Green Revolution that Combines a Second Green Revolution with a Blue Revolution. Remote Sens..

[3] FAO Food and Agriculture Organization of the United Nations. http://www.fao.org/sustainability/news/detail/en/c/1274219/.

[4] Matton N., Canto G.S., Waldner F., Valero S., Morin D., Inglada J., Arias M., Bontemps S., Koetz B., Defourny P. (2015). An Automated Method for Annual Cropland Mapping along the Season for Various Globally-Distributed Agrosystems Using High Spatial and Temporal Resolution Time Series. Remote Sens..

[5] Mtibaa S., Irie M. (2016). Land cover mapping in cropland dominated area using information on vegetation phenology and multi-seasonal Landsat 8 images. Euro-Mediterr. J. Environ. Integr..

[6] Boryan C., Yang Z., Mueller R., Craig M. (2011). Monitoring US agriculture: The US Department of Agriculture, National Agricultural Statistics Service, Cropland Data Layer Program. Geocarto Int..

[7] Oliphant A.J., Thenkabail P.S., Teluguntla P., Xiong J., Gumma M.K., Congalton R.G., Yadav K. (2019). Mapping cropland extent of Southeast and Northeast Asia using multi-year time-series Landsat 30-m data using a random forest classifier on the Google Earth Engine Cloud. Int. J. Appl. Earth Obs. Geoinf..

[8] Belgiu M., Csillik O. (2018). Sentinel-2 cropland mapping using pixel-based and object-based time-weighted dynamic time warping analysis. Remote Sens. Environ..

[9] Chakrabarti S., Cormier T., Malizia N., Potere D., Sulla-Menashe D., Zmijewski K., Friedl M. Mapping Cropland Extent by Asynchronous Fusion of Optical and Active Microwave Imagery. Proceedings of the IGARSS 2018—2018 IEEE International Geoscience and Remote Sensing Symposium.

[10] Potapov P., Turubanova S., Hansen M.C., Tyukavina A., Zalles V., Khan A., Song X.-P., Pickens A., Shen Q., Cortez J. (2022). Global maps of cropland extent and change show accelerated cropland expansion in the twenty-first century. Nat. Food.

[11] See L., Fritz S., You L., Ramankutty N., Herrero M., Justice C., Becker-Reshef I., Thornton P., Erb K., Gong P. (2015). Improved global cropland data as an essential ingredient for food security. Glob. Food Secur..

[12] Xiong J., Thenkabail P.S., Tilton J.C., Gumma M.K., Teluguntla P., Oliphant A., Congalton R.G., Yadav K., Gorelick N. (2017). Nominal 30-m Cropland Extent Map of Continental Africa by Integrating Pixel-Based and Object-Based Algorithms Using Sentinel-2 and Landsat-8 Data on Google Earth Engine. Remote Sens..

[13] Hendricks N.P., Er E. (2018). Changes in cropland area in the United States and the role of CRP. Food Policy.

[14] Rafif R., Kusuma S.S., Saringatin S., Nanda G.I., Wicaksono P., Arjasakusuma S. (2021). Crop Intensity Mapping Using Dynamic Time Warping and Machine Learning from Multi-Temporal PlanetScope Data. Land.

[15] Pan L., Xia H., Yang J., Niu W., Wang R., Song H., Guo Y., Qin Y. (2021). Mapping cropping intensity in Huaihe basin using phenology algorithm, all Sentinel-2 and Landsat images in Google Earth Engine. Int. J. Appl. Earth Obs. Geoinf..

[16] FAO Map Catalog—Food and Agriculture Organization of the United Nations. https://data.apps.fao.org/map/catalog/static/search?keyword=Crop%20intensity.

[17] Liu X., Zheng J., Yu L., Hao P., Chen B., Xin Q., Fu H., Gong P. (2021). Annual dynamic dataset of global cropping intensity from 2001 to 2019. Sci. Data.

[18] Gumma M.K., Thenkabail P.S., Maunahan A., Islam S., Nelson A. (2014). Mapping seasonal rice cropland extent and area in the high cropping intensity environment of Bangladesh using MODIS 500m data for the year 2010. ISPRS J. Photogramm. Remote Sens..

[19] Gumma M.K., Thenkabail P.S., Teluguntla P.G., Oliphant A., Xiong J., Giri C., Pyla V., Dixit S., Whitbread A.M. (2020). Agricultural cropland extent and areas of South Asia derived using Landsat satellite 30-m time-series big-data using random forest machine learning algorithms on the Google Earth Engine cloud. GIScience Remote Sens..

[20] Halder J. (2013). Land Suitability Assessment for Crop Cultivation by Using Remote Sensing and GIS. J. Geogr. Geol..

[21] Tran K.H., Zhang H.K., McMaine J.T., Zhang X., Luo D. (2022). 10 m crop type mapping using Sentinel-2 reflectance and 30 m cropland data layer product. Int. J. Appl. Earth Obs. Geoinf..

[22] Helman D., Lensky I.M., Tessler N., Osem Y. (2015). A Phenology-Based Method for Monitoring Woody and Herbaceous Vegetation in Mediterranean Forests from NDVI Time Series. Remote Sens..

[23] Ginn F. (2017). The International Encyclopedia of Geography: People, The Earth, Environment and Technology.

[24] Kong D., Zhang Y., Gu X., Wang D. (2019). A robust method for reconstructing global MODIS EVI time series on the Google Earth Engine. ISPRS J. Photogramm. Remote Sens..

[25] Li X., Shen R., Chen R. (2020). Improving Time Series Reconstruction by Fixing Invalid Values and its Fidelity Evaluation. IEEE Access.

[26] Li X., Wang L., Cheng Q., Wu P., Gan W., Fang L. (2019). Cloud removal in remote sensing images using nonnegative matrix factorization and error correction. ISPRS J. Photogramm. Remote Sens..

[27] Matsushita B., Yang W., Chen J., Onda Y., Qiu G. (2007). Sensitivity of the Enhanced Vegetation Index (EVI) and Normalized Difference Vegetation Index (NDVI) to Topographic Effects: A Case Study in High-Density Cypress Forest. Sensors.

[28] Kumari N., Saco P.M., Rodriguez J.F., Johnstone S.A., Srivastava A., Chun K.P., Yetemen O. (2020). The Grass Is Not Always Greener on the Other Side: Seasonal Reversal of Vegetation Greenness in Aspect-Driven Semiarid Ecosystems. Geophys. Res. Lett..

[29] Martín-Ortega P., García-Montero L.G., Sibelet N. (2020). Temporal Patterns in Illumination Conditions and Its Effect on Vegetation Indices Using Landsat on Google Earth Engine. Remote Sens..

[30] Xu L., Li B., Yuan Y., Gao X., Zhang T. (2015). A Temporal-Spatial Iteration Method to Reconstruct NDVI Time Series Datasets. Remote Sens..

[31] Padhee S.K., Dutta S. (2019). Spatio-Temporal Reconstruction of MODIS NDVI by Regional Land Surface Phenology and Harmonic Analysis of Time-Series. GIScience Remote Sens..

[32] Padhee S.K., Dutta S. (2020). Spatiotemporal reconstruction of MODIS land surface temperature with the help of GLDAS product using kernel-based nonparametric data assimilation. J. Appl. Remote Sens..

[33] You N., Dong J., Huang J., Du G., Zhang G., He Y., Yang T., Di Y., Xiao X. (2021). The 10-m crop type maps in Northeast China during 2017–2019. Sci. Data.

[34] D’Andrimont R., Verhegghen A., Lemoine G., Kempeneers P., Meroni M., van der Velde M. (2021). From parcel to continental scale—A first European crop type map based on Sentinel-1 and LUCAS Copernicus in-situ observations. Remote Sens. Environ..

[35] Yaramasu R., Bandaru V., Pnvr K. (2020). Pre-season crop type mapping using deep neural networks. Comput. Electron. Agric..

[36] Zheng B., Myint S.W., Thenkabail P.S., Aggarwal R.M. (2015). A support vector machine to identify irrigated crop types using time-series Landsat NDVI data. Int. J. Appl. Earth Obs. Geoinf..

[37] Ketchum D., Jencso K., Maneta M.P., Melton F., Jones M.O., Huntington J. (2020). IrrMapper: A Machine Learning Approach for High Resolution Mapping of Irrigated Agriculture Across the Western U.S. Remote Sens..

[38] Zhang C., Dong J., Xie Y., Zhang X., Ge Q. (2022). Mapping irrigated croplands in China using a synergetic training sample generating method, machine learning classifier, and Google Earth Engine. Int. J. Appl. Earth Obs. Geoinf..

[39] Sentinel-2. Marketplace—Google Cloud Console. https://console.cloud.google.com/marketplace/product/esa-public-data/sentinel2?pli=1.

[40] Dutta S., Rehman S., Chatterjee S., Sajjad H., Kumar Shit P., Pourghasemi H.R., Adhikary P.P., Bhunia G.S., Sati V.P. (2021). Chapter 3—Analyzing seasonal variation in the vegetation cover using NDVI and rainfall in the dry deciduous forest region of Eastern India. Forest Resources Resilience and Conflicts.

[41] Viana C.M., Oliveira S., Oliveira S.C., Rocha J., Pourghasemi H.R., Gokceoglu C. (2019). 29—Land Use/Land Cover Change Detection and Urban Sprawl Analysis. Spatial Modeling in GIS and R for Earth and Environmental Sciences.

[42] Liu R., Shang R., Liu Y., Lu X. (2017). Global evaluation of gap-filling approaches for seasonal NDVI with considering vegetation growth trajectory, protection of key point, noise resistance and curve stability. Remote Sens. Environ..

[43] Hao P., Tang H., Chen Z., Yu L., Wu M. (2019). High resolution crop intensity mapping using harmonized Landsat-8 and Sentinel-2 data. J. Integr. Agric..

[44] Li L., Friedl M.A., Xin Q., Gray J., Pan Y., Frolking S. (2014). Mapping Crop Cycles in China Using MODIS-EVI Time Series. Remote Sens..

[45] Sruthi E.R. Understand Random Forest Algorithms with Examples (Updated 2023). Analytics Vidhya. 17 June 2021. https://www.analyticsvidhya.com/blog/2021/06/understanding-random-forest/.

[46] Seif G. A Beginner’s Guide to XGBoost. https://towardsdatascience.com/a-beginners-guide-to-xgboost-87f5d4c30ed7.

[47] LSTM|Introduction to LSTM|Long Short Term Memory Algorithms. https://www.analyticsvidhya.com/blog/2021/03/introduction-to-long-short-term-memory-lstm/.

[48] Cornegruta S., Bakewell R., Withey S., Montana G. (2016). Modelling Radiological Language with Bidirectional Long Short-Term Memory Networks. arXiv.

[49] Pawar R. k-NN based Time Series Classification. https://towardsdatascience.com/k-nn-based-time-series-classification-e5d761d01ea2.

[50] Sole X., Ramisa A., Torras C. (2014). Evaluation of Random Forests on large-scale classification problems using a Bag-of-Visual-Words representation. Artificial Intelligence Research and Development.

[51] Gold O., Sharir M. (2018). Dynamic Time Warping and Geometric Edit Distance: Breaking the Quadratic Barrier. ACM Trans. Algorithms.

[52] Chen T., Guestrin C. XGBoost: A Scalable Tree Boosting System. Proceedings of the 22nd ACM SIGKDD International Conference on Knowledge Discovery and Data Mining.

[53] Hochreiter S., Schmidhuber J. (1997). Long Short-Term Memory. Neural Comput..

